# Triggering Receptor Expressed on Myeloid Cells-1 Signaling: Protective and Pathogenic Roles on Streptococcal Toxic-Shock-Like Syndrome Caused by *Streptococcus suis*

**DOI:** 10.3389/fimmu.2018.00577

**Published:** 2018-03-21

**Authors:** Li Han, Lei Fu, Yongbo Peng, Anding Zhang

**Affiliations:** ^1^State Key Laboratory of Agricultural Microbiology, College of Veterinary Medicine, Huazhong Agricultural University, Wuhan, China; ^2^Key Laboratory of Preventive Veterinary Medicine in Hubei Province, The Cooperative Innovation Center for Sustainable Pig Production, Huazhong Agricultural University, Wuhan, China; ^3^Key Laboratory of Development of Veterinary Diagnostic Products, Ministry of Agriculture, Wuhan, China; ^4^International Research Center for Animal Disease, Ministry of Science and Technology, Wuhan, China; ^5^Institute for Medical Biology, Hubei Provincial Key Laboratory for Protection and Application of Special Plants in Wuling Area of China, College of Life Sciences, South-Central University for Nationalities, Wuhan, China

**Keywords:** triggering receptor expressed on myeloid cells-1, streptococcal toxic-shock-like syndrome, *Streptococcus suis*, inflammation, cytokine storm

## Abstract

*Streptococcus suis* infections can cause septic shock, which is referred to as streptococcal toxic-shock-like syndrome (STSLS). The disease is characterized by a severe inflammatory response, multiple organ failure, and high mortality. However, no superantigen that is responsible for toxic shock syndrome was detected in *S. suis*, indicating that the mechanism underlying STSLS is different and remains to be elucidated. Triggering receptor expressed on myeloid cells-1 (TREM-1), belonging to the Ig superfamily, is an activating receptor expressed on myeloid cells, and has been recognized as a critical immunomodulator in several inflammatory diseases of both infectious and non-infectious etiologies. In this review, we discuss the current understanding of the immunoregulatory functions of TREM-1 on acute infectious diseases and then highlight the crucial roles of TREM-1 on the development of STSLS.

## Introduction

*Streptococcus suis* is a major swine pathogenic bacterium, and it is also a severe threat to human health (1–4). Since the first reported case of *S. suis*-induced meningitis in humans in Denmark in 1968, more than 1,600 human infection cases have been reported in the world (5, 6). In addition, *S. suis* has also been recognized as the leading and second cause of adult meningitis in Vietnam and Thailand, respectively (1, 7, 8). For a long time, *S. suis* infections in humans have remained sporadic and mainly affect individuals who have closely contacted with pigs or pig-derived products (9–11). However, the two large-scale outbreaks in China (12, 13) and human cases without a history of animal contact (14, 15) have modified opinion regarding the threat of this pathogen to humans.

*Streptococcus suis* infections in humans normally produce meningitis, endocarditis, cellulitis, peritonitis, arthritis, pneumonia, and occasionally septic shock, and the pooled case-fatality rate is 12.8% (1, 16, 17). Now, special attention is given to the largest outbreak in China in 2005, which caused 38 deaths among 204 human infections. Of the 38 deaths, 37 were caused by septic shock, which is designated as “streptococcal toxic-shock-like syndrome (STSLS)” (12). Unfortunately, 63% of STSLS patients died even after treatment with antibiotics (18), and STSLS is characterized by high serum levels of IFN-γ, TNF-α, IL-8, IL-12, and IL-1β, termed “cytokine storm” (19). However, no superantigen that is responsible for toxic shock syndrome was detected in *S. suis* (12), indicating that the mechanism underlying STSLS is different from that of toxic shock syndrome.

High levels of systemic pro-inflammatory cytokines was an important pathological cause for sudden death or meningitis induced by *S. suis* infection (20). Besides, the IFN-γ response was also confirmed to be responsible for causing high mortality of STSLS (21). These experiments suggested that inhibition of the exaggerated inflammatory response could improve the outcome of STSLS. However, these findings seemed conflict with a previous study that pre-administration of IL-1β increased neutrophil and monocyte numbers and bactericidal activity, and then facilitate to control *S. suis* challenge (22). Therefore, inflammatory response may play complicate roles during *S. suis* infection.

Since its discovery in 2000, triggering receptor expressed on myeloid cells-1 (TREM-1) has been described as a critical immunomodulator in several inflammatory disorders (23). Infection with *S. suis* also induced TREM-1 expression (24), indicating the signaling involved in this infectious disease. Here, we present the current progresses of the immunoregulatory functions of TREM-1 on acute infectious diseases and highlight the essential roles of TREM-1 on the development of STSLS.

## Function of TREM-1 on the Development of Infectious Diseases

Triggering receptor expressed on myeloid cells-1 was firstly identified on lipopolysaccharide (LPS)-stimulated neutrophils and monocytes (25), and then confirmed to be highly expressed on granulocytes, DCs, and natural killer cells and lowly expressed on T and B cells (26). TREM-1, belonging to the Ig superfamily, is a cell surface-activating receptor with a single extracellular V-type Ig-like domain, a transmembrane region containing charged lysine residues and a short cytoplasmic tail lacking signaling motifs (27, 28). TREM-1 can amplify toll-like receptor (TLR)-initiated responses against microbial challenges, enhancing the inflammatory response through interaction with an adaptor protein, DNAX-activating protein of 12 kDa (DAP12) (23, 25, 29). Due to its key role on enhancement of the inflammatory response, TREM-1 was recognized as an important regulator of innate immunity in sepsis (23, 30–33), septic shock (34–36), autoimmune arthritis (37), chronic inflammatory disorders (38), inflammatory bowel disease (39, 40), and corneal inflammation (41).

Despite these previous findings, the results regarding the requirement of TREM-1 for controlling of microbial infections are controversial. TREM-1 contributed to neutrophilic infiltration, induction of pro-inflammatory cytokines, and the disease severity, but it could not obviously affect pathogen clearance during *Leishmania major*, influenza virus or *Legionella pneumophila* infection (42). By contrast, TREM-1 played an important role in controlling dissemination of *Kelbsiella pneumoniae* and improvement of survival in a model of a *Klebsiella pneumoniae* liver abscess (43). Another example for the contribution of TREM-1 to killing pathogen was the infectious model on *Streptococcus pneumoniae* with *trem1/3^−/−^* mice (44) or agonistic TREM-1 antibody (45). TREM-1 was confirmed to play a role on secretion of cytokines and chemokines, neutrophils influx, clearance of *Streptococcus pneumoniae*, and improved survival (44, 45). Moreover, TREM-1/3 deficiency also increased local and systemic cytokine production, decreased the transepithelial migration of neutrophils into the airspace, and increased mortality during *Pseudomonas aeruginosa* infection (46). Therefore, these studies suggest that the roles of TREM-1-mediated immune responses to infection are very complicate.

## Soluble Form of TREM-1 (sTREM-1) and Infectious Disease

Apart from the membrane-bound form of TREM-1, a 27-kDa glycosylated peptide, corresponding to the sTREM-1, has been found in body fluids of infected individuals (30, 47). Two hypotheses have been proposed to explain the origin of sTREM-1: alternative splicing of TREM-1 mRNA (48) and proteolytic cleavage(s) of mature, membrane-anchored TREM-1 (49). With a general matrix metalloproteinase inhibitor, Gomez-Pina et al. demonstrated that metalloproteinases were responsible for shedding of the TREM-1 ectodomain through proteolytic cleavage of its long juxtamembrane linker (50).

The clinical significance of sTREM-1 has been confirmed in several studies in which sTREM-1 was detected in patients with chronic obstructive pulmonary disease (51), peptic ulcer disease (52), severe sepsis (53), septic shock (30), or inflammatory bowel disease (54). Now, sTREM-1 is recognized as a diagnostic and prognostic biomarker in patients with septic shock (55), neonatal sepsis (56), and *Streptococcus pyogenes*-induced sepsis (32).

At present, the function of sTREM is not fully understood. It is possible that sTREM-1 may negatively regulate receptor signaling through neutralization of the ligands, which is supported by the findings that the TREM-1 signaling could be significantly inhibited by a fusion protein containing the TREM-1 extracellular domain and human IgG1 Fc fragment (23) or the recombinant TREM-1 extracellular domain (57).

## Signaling for TREM-1 Expression

Triggering receptor expressed on myeloid cells-1 could be induced in response to various ligands, such as LPS (25, 58), bacteria (23, 41), and viruses (59, 60). In LPS-stimulated RWA264.7 cells, the transcription of TREM-1 was found positively and negatively regulated by NF-κB and PU.1 (61). In macrophages, LPS-induced TREM-1 expression was mediated, at least partly, by endogenous prostaglandins E2 followed by EP4 and cAMP, protein kinase A, p38 MAPK, and PI3K-mediated signaling (62). The expression of TREM-1 could also be inhibited by prostaglandins D2 and cyclopentanone prostaglandins PGJ2 and 15-dPGJ2, which was through activation of Nrf2 and inhibition of NF-κB. These provided a novel mechanism by which these prostaglandins show anti-inflammatory effects (63).

Based on the analysis of the *trem1* promoter, Hosoda et al. demonstrated that the cAMP response element (CRE) and NF-κB-binding site in the mouse TREM-1 promoter regulated the basal TREM-1 transcription positively and negatively, respectively (64). In addition, CRE and NF-κB possibly participated in the LPS-induced upregulation of TREM-1 promoter activity. AP-1 also seemed to be involved in the LPS-induced TREM-1 transcription through the interaction with phosphorylated c-fos/c-Jun (64).

Interestingly, TREM-1 expression in response to lipoteichoic acid is MyD88 dependent, and the expression induced by LPS is mediated by the TRIF signaling but not by MyD88, which suggest that signaling for TREM-1 induction is dependent on the specific TLR ligands (65).

## TREM-1 Ligands and Signaling

Activation of TREM-1 signaling is initiated when binding of the ligand to the receptor, which triggers the association and phosphorylation of the immunoreceptor tyrosine-based activation motif of the adaptor protein DAP12, resulting in the recruitment and activation of the non-receptor tyrosine kinase Syk. Syk, in turn, activates the downstream signaling molecules including PI3K, PLCγ, ERK1/2, and MAP kinases to induce the production of inflammatory chemokines and cytokines, such as IL-8 and myeloperoxidase (MPO), in neutrophils and IL-8, MCP-1, and TNF-alpha in monocytes (29, 66–68). In addition, TREM-1 also regulates macrophage survival through Bcl-2 (69), alters the dynamics of pulmonary IRAK-M expression, and improves host defense during pneumococcal pneumonia (45).

**Figure 1:**
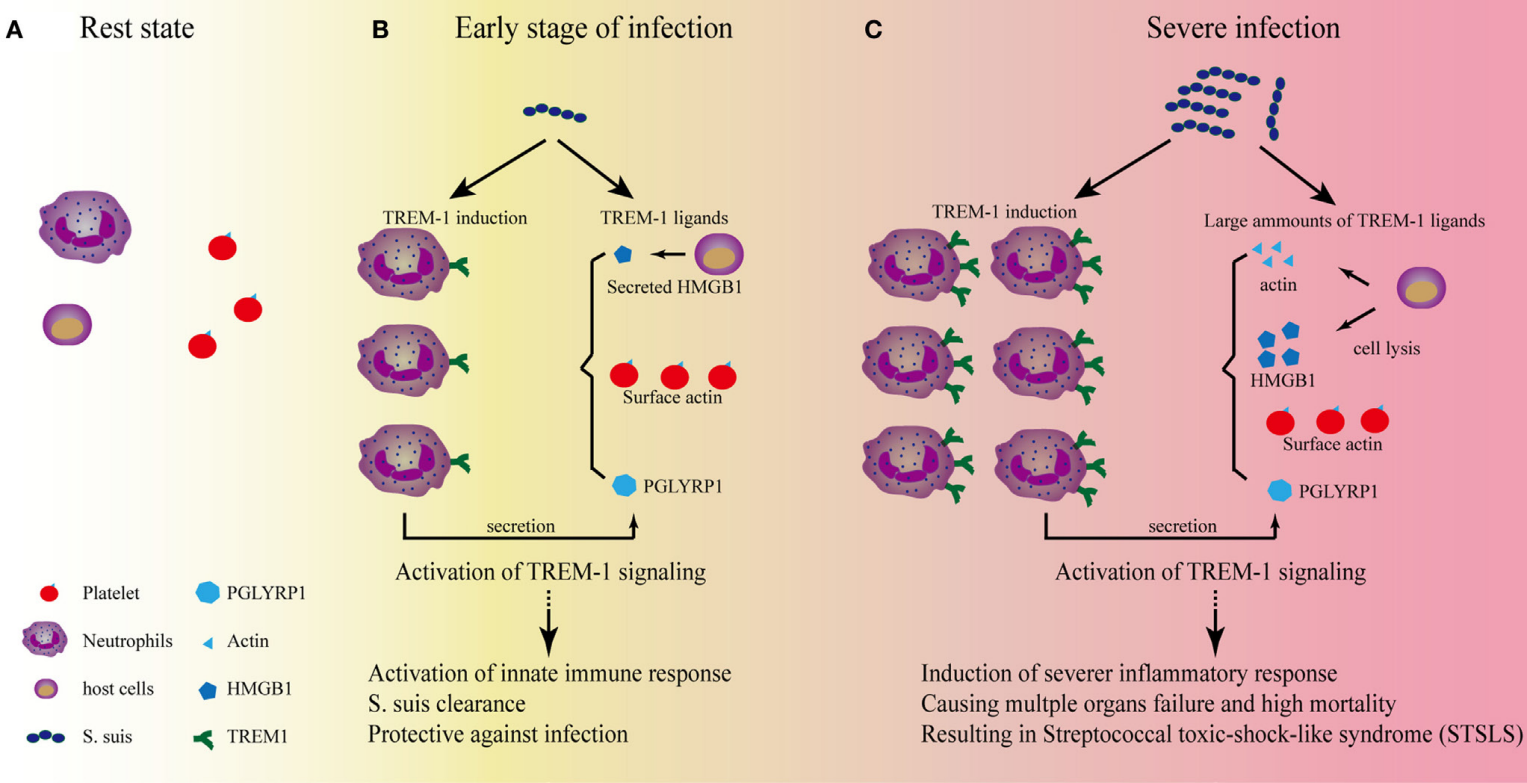
The role of triggering receptor expressed on myeloid cells-1 (TREM-1) signaling on the development of streptococcal toxic-shock-like syndrome (STSLS) caused by *Streptococcus suis*. **(A)** In the resting state, two reasons to confirm that TREM-1 signaling could not be activated by the surface actin on platelets: One reason is that TREM-1 expression is not induced; the other reason is that activation of TREM-1 on neutrophils by the surface actin on platelet requires the interaction of both cells, which is selectin/integrin dependent. Therefore, the signaling does not occur. **(B)** At the early stage of *S. suis* infection, TREM-1 expression is induced through various pattern-recognition receptors, such as toll-like receptor (TLR)2, TLR4, TLR6, and so on. The activated host cells could also secrete HMGB1 or peptidoglycan recognition protein 1 (PGLYRP1), which could serve as ligands for TREM-1 activation. In addition, the activated neutrophils could interact with platelets which could further provide surface actin for TREM-1 activation. The activation of TREM-1 signaling is essential for further activation of neutrophils and monocytes, which are important for bacterial clearance. At this stage, if *S. suis* could be significantly killed by these innate immune cells, the infection would be under control. **(C)** Severe infection would occur if the bacterial could resist the clearance. The Chinese epidemic *S. suis* strain has developed many strategies to resist the early killings, and the quick propagation of *S. suis* would provide more ligands for TLR activation to induce a significantly high level of TREM-1 expression. In addition, necrosis of host cells due to the infection of *S. suis* would provide much more ligands (such as actin and HMGB1) to activate TREM-1 signaling to cause severe inflammation. Ultimately, a TREM-1-mediated severe inflammatory response results in the cytokine storm, multiple organs failure, and high mortality—the characteristics of STSLS.

Identification of TREM-1 ligands is very important for understanding the nature of TREM-1 signaling. Gibot et al. first revealed that a TREM-1 ligand was induced on murine granulocytes during experimental peritonitis and sepsis (35). Interestingly, the surface glycoprotein of filoviruses was identified as a ligand for TREM-1 (70). Because endogenous signals released from necrotic cells could augment inflammatory responses through TREM-1, identification of the endogenous ligands would be more informative. HSP-70 and HMGB-1 from LPS-induced necrotic cell lysates might function as ligands for TREM-1, although the interaction between these proteins and TREM-1 was not confirmed in that study (71). Through screening hematopoietic cells for specific binding of a recombinant soluble fusion protein consisting of the extracellular domain of human TREM-1, Haselmayer et al. indicated that the natural ligand for TREM-1 was located on the surface of platelets (72). Considering the contribution of interaction between platelets and immune cells to the development of sepsis (73, 74), further identification of the ligands for TREM-1 activation in platelets was performed. Actin was identified as a TREM-1-interacting protein, and actin could activate inflammatory responses in a TREM-1-dependent manner (75). Since actin is a cellular cytoskeleton protein, there was a conflict about whether actin could be distributed on the cell surface. In fact, distribution of actin on the surface of platelets could be detected even in the resting state (76). Therefore, platelets did provide surface actin for TREM-1 recognition to activate signaling. In addition, HMGB1 was also confirmed as a TREM-1 ligand, which regulated Kupffer cell activation and development of hepatocellular carcinoma (77). The peptidoglycan recognition protein 1 (PGLYRP1) of neutrophils was also recognized as a functional ligand for TREM-1 (78). Until now, HMGB1, PGLYRP1, and extracellular actin have been identified as endogenous ligands for TREM-1 (75, 77, 78), which indicated that various proteins could be served as activate signal for TREM-1 signaling. Interestingly, all the identified endogenous ligands for TREM-1 were involved in the inflammatory conditions. PGLYRP1 could form homodimers for its antimicrobial activity and could be induced in response to the infection (79); HMGB1 and actin are the cellular proteins and could be released from the cells in inflammatory conditions (76, 80). The characteristics of these ligands for TREM-1 provided an image of how TREM-1 signaling can be activated to control infection or cause severe disease (Figure 1).

In the resting state (Figure 1A), two reasons to confirm that TREM-1 signaling could not be activated by the surface actin on platelets: one reason is that TREM-1 expression is not induced in normal conditions; the other reason is that activation of TREM-1 on neutrophils by the surface actin on platelet requires the interaction of both cells, which is selectin/integrin dependent (72).

By contrast, low-level stimulation could activate neutrophils or monocytes and then induce expression of TREM-1 through various pattern-recognition receptors (Figure 1B). Then, the active immune cells could interact with platelets through selectin/integrin dependent, which would further provide the condition for TREM-1 activation: the surface actin on platelets. In addition, the secreted PGLYRP1 and HMGB1 from host cells would also provide the endogenous signals for TREM-1 activation. This inflammatory condition mediated by TREM-1 signaling is required for some pathogen clearance (43, 44, 81).

However, if the pathogen could not be controlled by the inflammatory cells, the overwhelming stimulation might be presented. Then, TREM-1 expression would be induced significantly. Furthermore, the stimulation could further cause actin and HMGB1 to be released from the dying host cells, which would provide a large quantity of ligands for TREM-1 activation to cause progressive systemic inflammatory responses, resulting in severe inflammation.

## A Protective Role of TREM-1 on *S. suis* Infection

Through transcriptional analysis on the swine response to *S. suis* infection, Li et al. found that the expression of TREM-1 was induced and that a few inflammatory genes were also highly expressed (24). Using a recombinant TREM-1 extracellular domain or an agonistic TREM-1 antibody as an inhibitor or activator of signaling, Yang et al. found that blocking TREM-1 signaling could not improve the survival of mice experiencing *S. suis*-induced septic shock (81). This finding is inconsistent with the effects of blocking TREM-1 signaling on sepsis or septic shock caused by other pathogens (30–33, 35, 36). By contrast, they also found that the activation of TREM-1 signaling significantly improved the survival of mice infected with *S. suis* (81). These results indicated a protective role of TREM-1 on *S. suis* infection.

Furthermore, Yang et al. also noticed that TREM-1 blockage could intensify rather than inhibit the severe inflammatory response to *S. suis* infection, while activation could reduce inflammatory response (81). These results are confusing and give a contradictory function for TREM-1 signaling (23). However, the analysis on bacteria clearance indicated that the pro-inflammatory cytokine levels correlated well with the bacteria quantity *in vivo*, which suggested that blocking TREM-1 signaling may affect *S. suis* clearance, resulting in exacerbate inflammation (81).

Neutrophils played a very important role in controlling *S. suis* infection (82, 83), and an analysis indicated that TREM-1 signaling could significantly improve MPO level and neutrophils quantity in the blood during *S. suis* infection (81). Thus, the analysis further provided an explanation of how TREM-1 signaling provided a protective role of TREM-1 on *S. suis* infection: TREM-1 activation enhanced the activation of neutrophils and then contributed to the clearance of pathogen. Thus, TREM-1 blockage would inhibit inflammatory response and the activation of neutrophils, which would further reduce the clearance of *S. suis*. These would increase bacteria quantity and further cause severe inflammation to ultimately result in adverse outcomes of *S. suis* infection.

## Contribution of TREM-1 to STSLS

Triggering receptor expressed on myeloid cells-1 plays an essential role on *S. suis* clearance (81), and TREM-1 blockage alone cannot rescue the host from the infection. To directly evaluate the role of TREM-1 on causing severe inflammation, an inhibitor of TREM-1 signaling was used in the presence of antibiotics, although the treatment effectiveness on *S. suis* infection remains controversial (18). Treatment with ampicillin alone could kill bacterial efficiently and also reduce the inflammatory cytokine response; however, it cannot significantly improve survival rates (57). These findings are similar to the outcomes of the clinical treatment of pigs and humans during *S. suis* infection. However, killing the bacteria and blocking the TREM-1-mediated inflammatory response at the same time could effectively alleviate the severe inflammation and protect the host against epidemic *S. suis* infection (57). Thus, these results indicate that TREM-1 signaling also contributes to the development of severe inflammation and STSLS. Undoubtedly, TREM-1 blockage in the presence of effective antibiotics would be a valuable treatment for STSLS.

## Conclusion

*Streptococcus suis* infection may induce the expression of TREM-1 through various receptor, such as TLR2 (84–86), TLR4 (87), TLR6 (88), and so on, although the pattern-recognition receptor mainly responsible for STSLS remains to be identified (85, 89). At the early stage of infection (Figure 1B), TREM-1 recognizes the natural ligands (such as surface actin on the platelets) and activate neutrophils, which is essential for bacterial clearance (81). If the bacteria can be significantly killed by neutrophils, the infection will be under control. However, the Chinese epidemic strain has evolved many strategies to evade killing by host immune cells, such as resistance of phagocytosis (83) and acidic stress in lysosomes and endosomes (90), evading entrapment and killing by neutrophil extracellular traps (91, 92), resistance of complement-mediated killing (93, 94), and so on. If *S. suis* successfully resisted killing, the quick propagation of bacteria will provide much more ligands for the activation of pattern-recognition receptors to induce high levels of TREM-1 expression. In addition, necrosis of host cells due to the infection will also provide more ligands (such as actin and HMGB1) to activate TREM-1 signaling to cause severe inflammation. Ultimately, a TREM-1-mediated severe inflammatory response results in the cytokine storm, multiple organs failure, and high mortality—characteristics of STSLS (Figure 1C). Therefore, TREM-1 signaling plays protective and pathogenic roles on STSLS.

## Author Contributions

All authors listed have made a substantial, direct, and intellectual contribution to the work and approved it for publication.

## Conflict of Interest Statement

The authors declare that the research was conducted in the absence of any commercial or financial relationships that could be construed as a potential conflict of interest.
